# Buccal mucosa cell damage in individuals following dental X-ray examinations

**DOI:** 10.1038/s41598-018-20964-3

**Published:** 2018-02-06

**Authors:** Gang Li, Pan Yang, Shuai Hao, Wei Hu, Cheng Liang, Bing-shuang Zou, Xu-chen Ma

**Affiliations:** 1 Department of Oral and Maxillofacial Radiology, Peking University School and Hospital of Stomatology, Beijing, China; 2 Department of Orthodontics, Peking University School and Hospital of Stomatology, Beijing, China; 3 Department of Oral and Maxillofacial Surgery, Peking University School and Hospital of Stomatology, Beijing, China

## Abstract

The aim of the present study was to monitor genotoxic and cytotoxic effect of X-ray on exfoliated buccal mucosa cells and investigate the association between the effects and the accumulated absorbed doses of oral mucosa. 98 participants’ buccal mucosa cells were collected before and 10 days after different series of dental radiographs performed. Cytological preparations were successively dyed with the methods of Feulgen and fast-green, and analyzed under a light microscope. Micronuclei (MN)and other cells were scored. Accumulated absorbed dose of buccal mucosa was estimated with the method of anthropomorphic phantom and dosimeter chips. The dose rang was 0.18–3.54 mGy. A significant difference in the rate of MN cell was found before and after X-ray examinations (*P* = 0.008) as well as in the rates of Pyknotic (p < 0.001) and Karyolytic cell (p = 0.0021). When only the patients whose mucosa absorbed dose is lower than 1 mGy was analyzed, significant differences were not found except for Karyolytic cells (p = 0.0313). There was a correlation between the accumulated does and the change rate (ρ = 0.25, p = 0.0118). The frequency of micronuclei cells in buccal mucosa may be increased when a series of dental radiographs including a CBCT examination was performed.

## Introduction

Micronucleus (MN) is an anomaly structure in eukaryotic cells. It originates from chromosome fragments or whole chromosomes that lag behind at anaphase during nuclear division under physical and chemical factors. The MN index in rodent and human cells has become one of the standard cytogenetic endpoints and biomarkers used in genetic toxicology *in vivo* or *in vitro*. The increase of MN is a useful biomarker for the detection of human cancer risk in esophagus, bladder and oral tissue^1,2^. The MN assay can be performed in buccal and other exfoliated cells originating from rapidly divided epithelial tissue^3^. Researchers have managed to standardize the full procedure including evaluation process of Buccal Micronucleus Cytom Assay (BMCy) in order to assess genotoxic effect of carcinogenic factors, such as X-ray^4^. In this article, other cytome biomarkers, i.e. karyorrheix, pyknosis, karyolysis, condensed chromatin cells, binucleated cells, nuclear buds are also introduced for cytotoxic evaluation. MN and/or nuclear bud are a representative of DNA damage. Condensed chromatin, karyorrheix, pyknosis and karyolysis indicate apoptosis of a cell. Binucleated cell is an indication of cytokinetic defects and the frequency of basal cell shows a proliferative potential.

There have been a number of studies investigating the genotoxic effects of dental X-ray examinations by the use of BMCy^5–7^. In 2008, a study performed by Rebeiro *et al*. found that there was no significant difference for MN index in exfoliated buccal mucosa cells from 39 patients before and after a panoramic examination. In a following study, a series of radiographic examinations including panoramic, lateral and posteroanterior cephalometric radiographs were taken for 18 adolescents searching for orthodontic treatment, and the results indicate that the frequency of micronuclei cells was not significantly increased. In this study, the rates of pyknosis, karyolysis and karyorrhexis were also assessed for cytotoxicity and the results show a significant increase in these rates. With the introduction of cone beam computed tomography (CBCT) to dentistry, a study for CBCT was also performed and the results demonstrate a significant increase in the rates of pyknosis, karyolysis and karyorrhexis, but not for the rates of MN cells. These seem to indicate a safe use of the above mentioned X-ray examinations. However, we have to bear in mind that in these studies only one brand of the same type of dental X-ray machines was evaluated and radiation dose emitted from different brands of a same type of machine are quite different. For example, the effective dose obtained from panoramic machine Promax is about 24.3 μSv while for the panoramic machine Orthophos XG the effective radiation dose is only about 14.2 μSv, almost twice times lower than that of the Promax^8^. This makes the results from the mutagenicity studies that did not provide exact radiation doses hardly being compared and impossible to find any clue that indicates the relationship between genetic damage in buccal cells and radiation dose exposed to patient.

It is a well-known fact that radiation dose is accumulated. Clinically, patient is usually asked to take a series of radiographs including panoramic, lateral and posteroanterior radiographs in a very short period of time for the purpose of orthodontic or orthognathic treatment planning and/or prognosis evaluation. With the introduction of CBCT to dentistry, a CBCT scan for temporomandibular joint (TMJ) examination or a cranial-facial scan is occasionally included. In case that all the necessary radiographs including CBCT are acquired in a limited time, whether the radiation dose accumulated in such a short time would have a potential cancer risk for patient who undertakes such a series of radiographs? Since most of patients searching for orthodontic treatment are under 18 years old and youngster is more sensitive to ionizing radiation than adults, whether the potential cancer risk is increased for patient younger than 18 years old?

In the search of literature, we did not find any other study with regarding to the cellular damage of buccal mucosa cells in individuals exposed to such a series of radiographs within a limited time. Therefore, the aims of the present study were:

1. to monitor genotoxic effect of X-ray on exfoliated buccal mucosa cells during dental x-ray examinations;
2. to estimate the absorbed dose of irradiated buccal mucosa by the method of anthropomorphic phantom and thermoluminescent dosimeters;
3. to investigate the possible association between genotoxic and cytotoxic effect of X-ray on exfoliated buccal mucosal cells and the accumulated absorbed doses of oral mucosa during dental x-ray examinations.
4. to assess whether genotoxic and cytotoxic effect of X-ray on exfoliated buccal mucosal cells is more susceptible in patients younger than 18 years old.

## Materials and Methods

### Subjects

The subject included 98 patients who searched for orthodontic or orthognathic treatment in the hospital. Among the patients, 28 were male and 70 were female. The age ranges from 8 to 42 with an average age 23.63 ± 6.64. The criteria for the inclusion of patients were:

1. No habits of smoking and/or drinking;
2. No exposure to X-rays in recent three months;
3. No oral mucosa diseases;
4. No local stimulation factors;
5. would have dental X-ray examinations within 1 hour.

When not all the above inclusion criteria are met, a patient was excluded.

Altogether, 24 patients for orthognathic surgical treatment and 74 patients for orthodontic treatment were collected. Prior to X-ray examinations, patient individual information such as age, gender, medical history, radiographs exposed and the exposure parameters were recorded for later analysis. All the acquired radiographs were those necessary for treatment planning and not specific for the study.

An information consent form was signed by the participants or their guardians. The study was approved by the Institutional Review Board of Peking University School and Hospital of Stomatology. All methods were performed in accordance with the relevant guidelines and regulations.

### Dental X-ray examinations

One or several of the following dental X-rays were performed for individual patient: panoramic radiograph, lateral cephalometric radiograph, posteroanterior cephalometric radiograph, a CBCT scan for TMJ, a CBCT scan for the whole skull and a CBCT scan for maxilla. CBCT scans were performed with a DCT Pro scanner (VATECH&E-WOO Corporation, Seoul, Korea; 90 kVp, 5–7 mA, 24 s) or a NewTom VG (Quantitative Radiology, Verona, Italy; 110 kVp, 6.24–14.45 mAs). Panoramic radiograph, lateral radiograph and posteroanterior radiograph were performed with an all-in-one dental digital imaging system Orthopantomograph OP100 (Instrumentarium Imaging Corporation, Tuusula, Finland). The exposure parameters were 66 kVp, 4–10 mA, 17.6 s for the panoramic radiographs and 77 kVp, 12 mA, 0.5–1.0 s for the lateral radiographs, 77 kVp, 12 mA, 0.8–1.2 s for the posteroanterior radiographs.

### Cell collection and slides preparation

Exfoliated oral mucosa cells were collected immediately before dental X-ray examinations and 10 days later. After rinsing the mouth with tap water, cells were obtained by swabbing both left and right cheek mucosa of the patients with a moistened wooden spatula. Cells were transferred to a tube of buccal cell buffer (0.16% Tris-HCL, 0.12% EDTA, 3.72% sodium chloride), centrifuged three times (2000 rpm, 3 min), fixed in 3:1 methanol/acetic acid, homogenized for 5 min, dropped onto pre-cleaned slides and dried in air. Slides were successively stained with the method of Feulgen/fast green.

### Cytological observation

**Figure 1:**
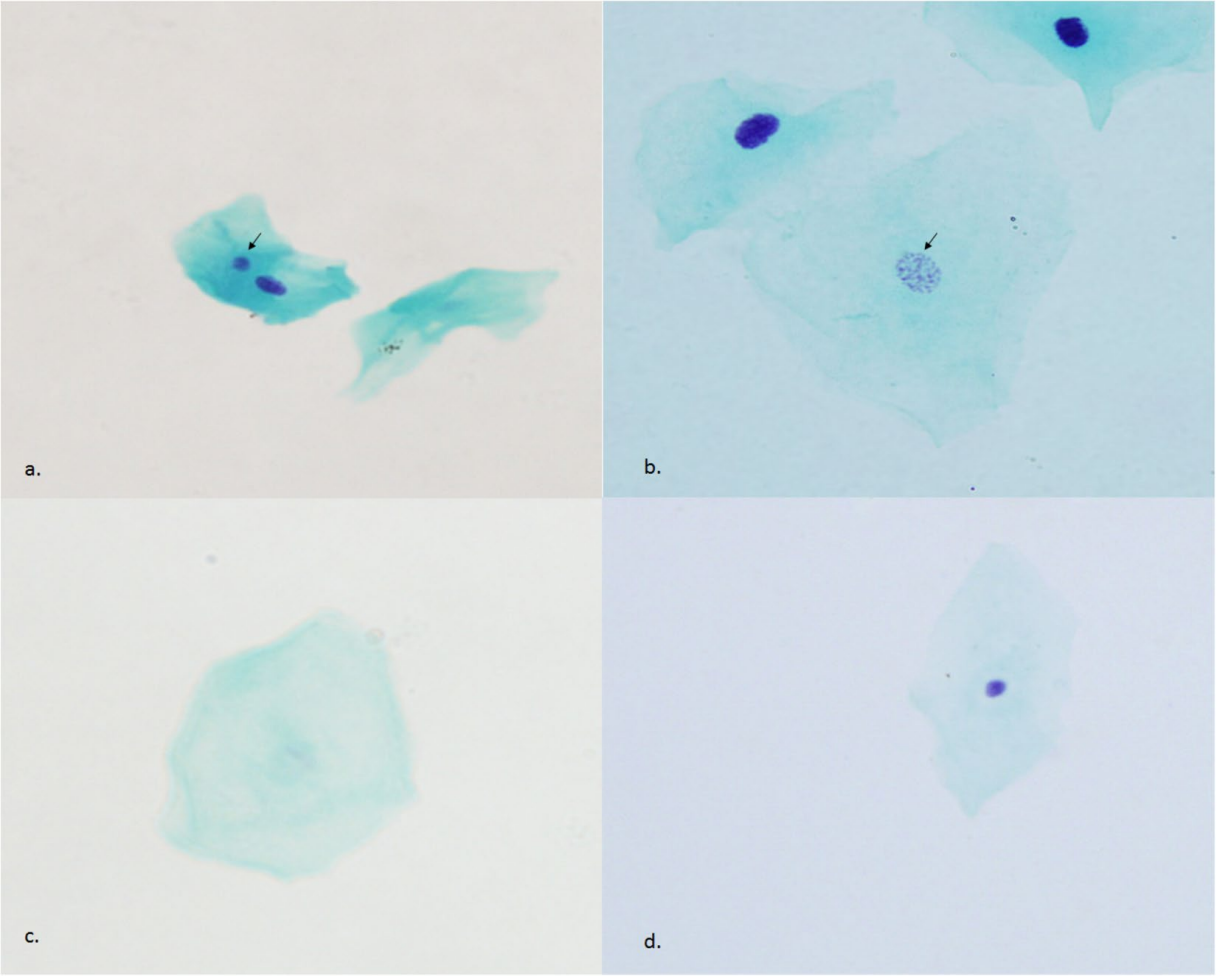
Photomicrographs of cells with (**a**) micronucleus (arrow), (**b**) karyorrhexis (arrow), (**c**) karyolysis and (**d**) pyknosis. Magnification x400.

Cytological observation was performed with a light microscope BX51 (Olympus corporation, Tokyo, Japan) at x400 magnification. The frequency of micronuclei cells was counted in 2000 cells and other types of cells such as basal cells, binucleated cells, condensed chromatin cells, karyorrhectic cells, pyknotic cells, karyolytic cells and cells with nuclear buds were scored in 1000 cells for each individual. All kinds of cells were scored according to the criteria described by Thomas P *et al*.^4^. Micronuclei cells as a parameter for DNA damage were distinguished on the basis of five characteristics: (a) be less than 1/3 diameter of the main nucleus; (b) be on the same plane of focus; (c) have the same color, texture and refraction as the main nucleus; (d) have smooth round or oval shape, and (e) be clearly separated from the main nucleus^9^. Sample images of the observed MN and other cells were shown in Fig. 1.

For intra-observer variability analysis, ten slides were randomly selected for observation of cell anomalies two month later.

To study whether the rate changes of different types of cells are really connected to dental x ray examinations, 8 patients were called back one and half year later and had the mucosa cells collected for cytogenetic observation again.

### Measurement of accumulated absorbed doses of oral mucosa

An human anthropomorphic phantom (ART-210, Radiology Support Device, Inc., Long Beach, CA, USA.) and thermoluminescent dosimeter (TLD) chips were used for the estimation of accumulated absorbed dose of oral mucosa. The phantom was with tissue equivalent X-ray attenuating characteristics and closely conforms to International Commission on Radiation Units and Measurements specifications^10^.

The accumulated absorbed doses of oral mucosa were measured at each of the nine protocols described in Table 1. Before the study, all dosimeters were calibrated using a Co-60 source. Detailed information regarding the measurements was described in a previous study^11^.

**Table 1:** Dental X-ray examinations and the corresponding accumulated absorbed doses of oral mucosa.

Radiographs	Absorbed dose (mGy)	N	Group
Panorama Panorama + Lateral radiograph	0.18 0.21	1 7	Low dose group
Panorama + Lateral + Frontal radiograph	0.24	2	n = 10
Cranifacial CBCT image (NewTom VG)	1.58	46	
Cranifacial CBCT image (NewTom VG) + Panorama + Lateral radiograph	1.79	1	
Cranifacial CBCT image (NewTom VG) + Panorama + Lateral + Frontal radiograph	1.82	1	
TMJ CBCT images (DCT-Pro)	2.62	2	
Frontal + TMJ CBCT images (DCT-Pro)	2.64	1	
Panorama + Lateral + TMJ CBCT images (DCT-Pro)	2.83	9	
Panorama + Lateral + Frontal + TMJ CBCT images (DCT-Pro)	2.86	25	Large dose group
Maxilla CBCT image (DCT-Pro)	3.4	1	n = 88
Panorama + Lateral + Craniofacial CBCT images (DCT-Pro)	3.51	1	
Panorama + Lateral + Frontal + Craniofacial images (DCT-Pro)	3.54	1	
Sum	n = 98		

CBCT: Cone Beam CT.

### Statistical analysis

Software package SPSS v16.0 for windows (SPSS, Chicago, IL, USA) was employed for the statistical analysis. Differences between frequencies of micronuclei cells, basal cells, binucleated cells, condensed chromatin cells, karyorrhectic cells, Pyknotic cells, Karyolytic cells and cells with nuclear buds before and after X-ray examinations were analyzed by Wilcoxon signed rank test. Since the basic images required for an orthodontic or orthognathic surgical treatment are 2 dimensional planar images, the absorbed doses were accordingly divided into two groups, low and large dose groups, for further analysis. Wilcoxon signed rank test was also used to analyze different cell rates before and after X-ray examinations in both the large and the low dose groups. For the analysis of correlation among different absorbed dose levels and changes of cell rates, Spearman rank correlation was employed. The changes of cell rates is the difference in the counted cell rates before and after radiographic examination (change of cell rates = Cell rate after X-ray examinations – Cell rate prior to X-ray examinations). According to age 18, the patients was divided into two groups, that is, in one group the patients was younger than 18 years old and in the other group the patients were older or equal to 18 years old. To investigate the age effect on the observed cell anomalies, the change rates obtained from both the low and large dose group were analyzed by Mann-Whitney test. For intra-observer variability, one-way ANOVA was employed. Differences were considered to be statistically significant when *P* < 0.05.

## Results

### Accumulated absorbed doses of oral mucosa

The accumulated absorbed doses of oral mucosa measured with the phantom were listed in Table 1. When only 2 dimensional planar radiographs were taken, the accumulated absorbed doses were no more than 1 mGy.

### Alterations of MN and other cells

The average occurrence rates, lower and upper quartile and p values of MN cells and other cells before and after X-ray examinations for all the 98 participants, for the large dose group of patients and only for the low dose group of patients was shown in Tables 2–4, respectively. Tables 2 and 3 demonstrates a significant difference for the frequency of MN cells as well as pyknotic and karyolitic cells before and after x-ray examinations. When only the low dose group of samples were analyzed, a significant difference between the frequencies of prior to and after X-ray examinations was not found for the MN cells but for the Karyolytic cells (P = 0.0313, Table 4).

**Table 2:** Mean, lower and upper quartile of different cells for all the 98 patients and the related P values obtained from Wilcoxon signed rank test.

Cell	Before X-ray examination (‰)		After X-ray examination (‰)		P value
	mean	25–75%	mean	25–75%	
Micronuclei cell*	0.38	(0 to 1)	0.60	(0 to 1)	0.008
Basal cell	11.81	(8 to 15)	11.08	(6 to 15)	0.0755
Bi-nucleated cell	7.73	(5 to 10)	7.93	(5 to 10)	0.8001
Condensed chromatin cell	5.72	(3 to 8)	6.03	(3 to 8)	0.0907
Karyorrhectic cell	1.74	(0 to 3)	2.10	(0 to 3)	0.5112
Pyknotic cell*	1.26	(0 to 2)	2.4	(0 to 4)	<0.001
Karyolytic cell*	46.38	(27 to 64)	57.92	(26 to 68)	0.0021
Nuclear buds	0.06	(0 to 0)	0.11	(0 to 0)	0.3629

*Significant difference at p < 0.05.

**Table 3:** Mean, lower and upper quartile of different cells for large dose patients and the related P values obtained from Wilcoxon signed rank test.

Cell	Before X-ray examination (‰)		After X-ray examination (‰)		P value
	mean	25–75%	mean	25–75%	
Micronuclei cell*	0.41	(0 to 1)	0.65	(0 to 1)	0.0098
Basal cell	11.96	(8 to 15)	11.10	(6 to 15)	0.0567
Bi-nucleated cell	7.87	(5 to 10)	7.99	(5 to 10)	0.8977
Condensed chromatin cell	5.65	(3 to 8)	6.30	(3 to 8)	0.4195
Karyorrhectic cell	1.69	(0 to 3)	2.16	(0 to 3)	0.3277
Pyknotic cell*	1.39	(0 to 2)	2.65	(1 to 4)	0.0001
Karyolytic cell*	46.81	(28 to 70)	62.87	(32 to 90)	0.0003
Nuclear buds	0.05	(0 to 0)	0.12	(0 to 0)	0.2407

*Significant difference at p < 0.05.

**Table 4:** Mean, lower and upper quartile of different cells for low dose patients and the related P values obtained from Wilcoxon signed rank test.

Cell	Before X-ray examination (‰)		After X-ray examination (‰)		P value
	mean	25–75%	mean	25–75%	
Micronuclei cell	0.10	(0 to 0)	0.20	(0 to 0)	1.0000
Basal cell	10.50	(7 to 14)	10.9	(6 to 16)	0.9883
Bi-nucleated cell	6.50	(4 to 9)	7.40	(7 to 10)	0.7168
Condensed chromatin cell	6.40	(4 to 9)	3.60	(2 to 4)	0.0859
Karyorrhectic cell	2.2	(0 to 3)	1.50	(0 to 2)	0.2500
Pyknotic cell	0.1	(0 to 0)	0.1	((0 to 0)	1.0000
Karyolytic cell*	24.2	(10 to 36)	12.90	(9 to 14)	0.0313
Nuclear buds	0.1	(0 to 0)	0.00	(0 to 0)	1.0000

*Significant difference at p < 0.05.

### Absorbed doses and cellular abnormalities

Table 5 demonstrates the association of different types of cells at different accumulated absorbed dose levels. A significant correlation for the micronuclei cell and the absorbed doses (*r*_*s*_ = 0.250, P = 0.0118) was observed but not for other types of cells.

**Table 5:** Spearman rank correlation coefficient and the related P values for the average change rate of different cells at different absorbed dose levels.

	Average change rate (‰)		Correlation coefficient	P value
	0~1 mGy	1~4 mGy		
Micronuclei cells	0.10	0.24	0.250*	0.0118
Basal cells	0.40	−0.86	−0.145	0.1476
Bi-nucleated cells	0.90	0.12	−0.010	0.9193
condensed chromatin	−2.80	0.65	−0.047	0.6393
Karyorrheix cells	−0.70	0.47	0.101	0.3168
Pyknosis cells	0.00	1.25	0.030	0.7675
Karyolysis cells	−11.3	14.05	0.018	0.8601
Nuclear buds	−0.1	0.07	0.098	0.3278

*Denotes a significant difference at p < 0.05.

### Age and the cellular abnormality alterations

In the low dose group there were 8 patients under 18 years old while in the large dose group the number of patients younger than 18 was 9. Since radiation dose has an impact on the frequency of different types of cells observed (Tables 3 and 4), the effect of age was investigated separately in the large and the low dose group to avoid the bias from radiation dose. There were no significant differences between the patients younger and older than 18 years old in both of the large (P = 0.118~0.729) and low dose (P = 0.080~1.0) groups.

### Intra-observer variability and verification of the effect of dental x-ray examinations

The intra-observer variance was not significant (P = 0.065~0.773).

Verification of the effect of dental X-ray examinations on different types of oral mucosa cells were shown in Table 6. Although no significant differences were found for the different types of oral mucosa cells among the eight patients with and without x-ray examinations, the frequency of average micronuclei cells was increased from 0.38 to 0.75 in the group with X-ray examinations while in the group without X-ray examinations, this cell frequency was down to 0.25 from 0.5.

**Table 6:** Mean, lower and upper quartile and p values of different cells for the 8 patients.

	Cell	First sampling (‰)		Second sampling (‰)		P value
		mean	25%–75%	mean	25%–75%	
Without X-ray examination	Micronuclei cell	0.5	(0 to 1)	0.25	(0 to 0)	0.75
	Basal cell	8.31	(5 to 11)	9.25	(7 to 11.5)	0.67
	Bi-nucleated cell	11.31	(8 to 13)	8.75	(7.5 to 10)	0.13
	Condensed chromatin cell	6.81	(4.5 to 9)	9.94	(4.5 to 8)	0.87
	Karyorrhectic cell	1.68	(1 to 2.25)	2.44	(1.5 to 2.25)	0.66
	Pyknotic cell	2.75	(1 to 4)	2.31	(0.75 to 3)	1.00
	Karyolytic cell	35.5	(25 to 47)	45.81	(32 to 60)	0.15
	Nuclear buds	0.06	(0 to 0)	0.06	(0 to 0)	1.00
With X-ray examination	Micronuclei cell	0.38	(0 to 1)	0.75	(0 to 1)	0.25
	Basal cell	15.13	(9.5 to 15)	10.13	(3.5 to 15.5)	0.16
	Bi-nucleated cell	10.13	(6.5 to 14)	9.75	(8 to 11.5)	0.88
	Condensed chromatin cell	7.25	(5 to 9.5)	6.38	(3 to 10)	0.74
	Karyorrhectic cell	2	(0.5 to 3.5)	3.38	(1 to 5.5)	0.38
	Pyknotic cell	1.63	(0 to 3)	1.88	(1 to 3)	0.81
	Karyolytic cell	37.25	(19 to 38)	35.38	(12 to 52.5)	1.00
	Nuclear buds	0.13	(0 to 0)	0.25	(0 to 0)	1.00

*Significant difference at p < 0.05.

## Discussion

This study demonstrates that the observed frequency of MN cells was significantly increased after a series of dental x-ray examinations. However, when only the low dose group was analyzed, the observed frequency of MN cells was not significantly different. This is in line with the previous studies^5,6,12–14^, which demonstrated that 2-dimentional planar dental X-ray examination may not relate to an increased risk of gene mutagenesis. Previous studies show that the frequency of karyorrheix cells, pyknotic cell and karyolitic cell was significantly increased after 2-dimensional planar radiographic examination^5,6,13,14^. However, in the present study only the frequency of karyolytic cells was significantly increased after 2-plananr radiographic examinations, that is, in the low dose group of the studied samples. This may relate to the relatively small sample size in the present study. For the large dose group, the significant differences between the frequency of pyknotic and karyolitic cells before and after the dental X-ray examinations were also observed. These results verify the hypothesis that dental X-ray examination promote cytotoxicity of oral mucosa cells. Compared to the previous studies where only 2-planar radiographs were employed^5,6,13,14^, the doses presented in the large dose group of the present study is relatively large (about ten times), this may explain why the observed frequency of MN cells was significantly increased in the large dose group.

To further disclose the association between genotoxic and cytotoxic effect of X-ray on exfoliated buccal mucosal cells and the accumulated absorbed doses during dental X-ray examinations, a change of the observed cell rates before and after dental radiographic examinations was employed. The reason why to use changes of cell rates for analysis is due to the consideration that individuals have different ability in producing different types of cells. Although the results indicate a correlation between the change rate of micronuclei cells and the accumulated absorbed doses of oral mucosa, the correlation coefficient was only 0.250. This result may be explained as week relationship between the two variables. A large sample may need further to disclose the relationship.

An interesting finding of the study is that the accumulated absorbed dose was increased dramatically when CBCT examination was performed. This indirectly identifies the results from other studies that the radiation dose of CBCT is much higher than those of conventional dental X-rays^15,16^.

Chromosomal damage that leads to the formation of micronuclei takes place in the basal layer of the epithelial tissue, where cells undergo mitosis. Rapid turnover of epithelial tissues brings the cells to the surface, where they exfoliate. As a result, the maximal rate of micronuclei formation in exfoliated cells is observed between 1 and 3 weeks after exposure to genotoxic agent. This is also true for other types of cells observed in the study^1,3,4^. For this reason, a period of 10 days after X-ray examination was adopted.

It is suggested that there are more cancer risk from radiation in youngsters than in adults, especially for those younger than 18 years old^17^. To monitor the age effect, the patients included in the present study was divided into two groups according to the age 18. The results show that no statistically significant differences were observed between the two groups of patients with respect to the genotoxic and cytotoxic effects of dental X-ray examinations on oral mucosa cells. This may be due to the fact that the number of patients younger than 18 years old in the study was relatively small.

To verify the association of the elevated rate changes of different cells with X-ray exposures, eight patients of the studied sample were called back one and half year later. The same test procedure were performed without any dental X-ray exposures to the patients. Although the results did not display any statistically significant differences, an elevated frequency of micronuclei cells was observed. This may identify that the increased rate changes of micronuclei cells is a result from dental X-ray examinations.

The limitation of the present study was the phantom used for the estimation of absorbed doses. The phantom was a representative of a male with a body height about 170–175 cm. This makes the absorbed doses diverge from the actual doses absorbed by the real patients. However, when considering the fact that only 9 out of 88 patients were younger than 18 years old in the large dose group and the results from the low dose group was similar to those from the other studies, it is reasonable to believe that the diverged absorbed doses was in an acceptable range.

## Conclusion

An increase in the frequency of micronucleus cells, a biomarker of genotoxic effect, as well as pyknotic and karyolitic cells from the exfoliated buccal mucosa was observed when a series of dental radiographs including a CBCT examination was performed. Age did not play a role in the genotoxic and cytotoxic effects of dental x-rays on oral mucosa cells.
